# Managers’ sick leave recommendations for employees with common mental disorders: a cross-sectional video vignette study

**DOI:** 10.1186/s40359-023-01086-6

**Published:** 2023-02-24

**Authors:** Jenny Hultqvist, Gunnel Hensing, Stephen Stansfeld, Monica Bertilsson

**Affiliations:** 1 Department of Health and Rehabilitation, Institute of Neuroscience and Physiology, Gothenburg University, PO Box 455, 405 30 Gothenburg, Sweden; 2 School of Public Health and Community Medicine, Gothenburg University, PO Box 463, 405 30 Gothenburg, Sweden; 3 Centre for Psychiatry, Queen Mary University of London, Mile End Road, London, E1 4NS Great Britain UK

**Keywords:** Depression, Employee, Managers, Sick leave, Video vignette study

## Abstract

**Background:**

To better understand the initial phases of sickness absence due to common mental disorders (CMD), the aim of the present video vignette study was to test the following three hypotheses: (1) Managers who have negative attitudes towards employees with CMD will not recommend sick leave. (2) Managers with experience of CMD recommend sick leave to a significantly higher extent than managers lacking this experience. (3) Managers with previous experience of recommending sick leave for people with CMD will recommend sick leave to a significantly higher extent also based on the vignettes.

**Methods:**

An online survey, including a CMD-labelled video vignette, was sent to 4737 Swedish managers (71% participated, *n*  = 3358). For aims (1) and (2), a study sample consisting of 2714 managers was used. For aim (3), due to the design of the survey questions, a subsample (*n*  = 1740) was used.

**Results:**

There was no significant association between negative attitudes towards employee depression and managers’ recommendation of employee sick leave with the vignette case. The bivariate analysis showed that personal experience of CMD was associated with managers’ recommendation of employee sick leave. In the adjusted regression model, it became non-significant. Previous experience of recommending sick leave to one employee and to several employees was associated with recommending sick leave, also when adjusting for gender, level of education, years of managerial experience, and management training on CMDs

**Conclusions:**

The likelihood of a manager recommending sick leave after watching a CMD-labelled video vignette was higher if the manager had previous experience of this situation in real life. This study highlights the importance of including managerial behaviours and attitudes to better understand sick leave among employees with CMD.

**Supplementary Information:**

The online version contains supplementary material available at 10.1186/s40359-023-01086-6.

## Background

Managers’ actions can play a role in the process of return to work for employees who are off sick with common mental disorders (CMD), predominantly depression and anxiety [1]. Less is known about managers’ behaviour and actions at the start of the process of CMD-related sickness absence, i.e., when an employee goes on sick leave due to CMD. Sickness absence with CMD is a complex matter involving the sick-listed employee, the manager, the health care, and the social insurance agency. Of these stakeholders, the managers’ actions are the least explored despite their presumed important role [2, 3]. Managers are at the frontline of the daily operations of the workplace, and can take early, and possible preventive, action when a subordinate shows signs of work instability due to CMD [4, 5]. One possible action could be recommending sick leave. Several factors may argue for, or against, managers’ recommendation for sick leave, and contemporary knowledge largely rests upon qualitative studies. To address this knowledge gap, the present study was designed as a survey, including a CMD-labelled video vignette, with the aim of understanding managers’ recommendation for sick leave and what the determinants are for this.

Occupational health and safety laws and regulations are common in many western societies. These regulations hold the employers responsible for protecting the health and safety of their employees [6]. This is also true for Sweden where the present study was performed [7]. Managers and supervisors have a crucial role in supporting employees with CMD and the provision of such support and help has been shown to be associated with less CMD-related sick leave days [8]. Even so, research suggests that managers can have negative attitudes towards CMD, and that these attitudes can affect managers’ behaviour and actions regarding hiring decisions and supportive practices [9, 10]. In addition, a study by Telwatte et al. [11] reported that managers were less likely to approve work adjustments for employees with CMD because they perceive them as less reasonable and legitimate compared with work adjustments for employees with other illnesses.

Another issue related to managers’ attitudes and actions regarding employees with CMD is the gender of the worker with CMD [8]. With respect to prevalence, women are at greater risk than men of developing CMD [12], and managers might assume that CMD is mainly due to gender and originating from personal factors. CMD may then be understood as a disorder that is not work-related but related to the person. Accordingly, it should be managed in the private sphere, for instance by taking sick leave, rather than through supportive measures in the work environment [9]. Furthermore, male managers have been found to hold more negative attitudes towards CMD compared with female managers [10, 13]. Conversely, managers who have personal experience of CMD, have a close friend or family member with CMD, or have experience of an employee with CMD have been shown to have less negative attitudes [10, 13].

A further issue with regard to managers’ role and possible involvement in subordinates’ sickness absence is their responsibility for work productivity. This could influence managers to recommend sickness absence because of employees’ reduced work performance, which has been reported to affect productivity negatively [14, 15]. Another reason to recommend sickness absence could be that the managers are concerned about deterioration in an employee’s mental health and safety issues at the workplace. To sum up, several factors and characteristics may argue for or against managers’ recommendation for CMD-related employee sick leave, including attitudes towards CMD, personal experience of CMD, gender of the employee, manager’s gender, and work-related issues.

Managers’ behaviour and actions in relation to employees with CMD can also be understood from a theoretical perspective, for example, within an attitude-behaviour framework or model [16, 17]. In these models, the frequency of past behaviour is acknowledged to play a significant role in the prediction of future actions/behaviour. Danner et al. [18] showed that the stability of the context in which the behaviour is performed plays a crucial role in the establishment of habits, i.e. predictive behaviour. A manager’s workplace context could be considered stable, for instance, in time and place, work sector and number of employees. Hypothetically, we can thus assume that the actions of managers in relation to employees with CMD in the context of their workplace predict managers’ future actions and behaviours. Accordingly, previous experience of recommending employee sick leave as well as workplace contextual factors should be addressed when exploring managers’ recommendations of employee sick leave.

The use of case vignettes is a common method in experimental research [19]. Case vignettes are short descriptions of persons or situations to elicit attitudes, beliefs or behaviours of respondents with respect to the scenario presented [19]. Among the many advantages of using vignettes, the possibility of anchoring survey questions into a realistic context and the possibility for random assignment of different vignettes have been highlighted [19]. In the choice between a written and a filmed vignette, the filmed vignette has the advantage of increasing the participants’ adherence to the vignette and limiting associations to other experiences elicited by written vignettes, thereby increasing reliability. Research applying vignettes to explore differences in attitudes to support and sick leave due to the gender of the protagonist with CMD have shown mixed results. In a British study [20], 1218 participants from the general population were randomized to receive a text vignette of a male or female character experiencing CMD. The results showed that male participants, compared with female participants, were less likely to recommend help for the male vignette. A Norwegian study [21] explored attitudes towards sick leave among managers and non-managerial employees using written vignettes describing different medical conditions. The results showed that the gender of the protagonist in the vignette had no effect on the managers’ evaluations of the appropriateness of sick leave. Another study from Norway [22] used four video vignettes to explore correspondence of 514 stakeholders’ (*n* = 107 managers) assessments of health, work capacity and sick leave certification. One of the four video vignettes presented a mental health-related situation. The results regarding this vignette showed that the managers were less likely to assess poor health and reduced work capacity, and more likely to suggest full time work and partial sick leave compared with general practitioners.

With the purpose to better understand manager’s role in the initial phases of CMD-related sickness absence, the aim of the present video vignette study was to test the following hypotheses:

- *Hypothesis 1* Managers who have negative attitudes towards employees with CMD will not recommend sick leave.
- *Hypothesis 2* Managers with experience of CMD recommend sick leave to a significantly higher extent than managers lacking this experience.
- *Hypothesis 3* Managers with previous experience of recommending sick leave for people with CMD will recommend sick leave to a significantly higher extent also based on the vignettes.

## Methods

### Study design

This cross-sectional video vignette study is part of the project “Managers’ perspective—the missing piece” within the research programme “New ways—mental health at work”. Data on attitudes, knowledge and strategies among Swedish managers were collected through a web-based questionnaire in 2017. CMDs were formulated as depression and anxiety disorders throughout the questionnaire to represent the two main categories of CMD diagnoses. The study incorporated a CMD-labelled video vignette of approximately 2 min duration. Half of the participants watched a video vignette with a female protagonist, and the other half watched a video vignette with a male protagonist, both aged about 35–40 years and played by actors. The setting was that the person had asked for an appointment with the manager and the conversation took place in the manager’s office. The protagonists then shared their problems in handling their work situation with the manager. The script for the video vignettes was developed within the project group where three researchers had in-depth knowledge based in qualitative research about capacity to work while depressed and anxious [23, 24]. Two of the researchers had long experience as managers and several researchers had extensive clinical experience in return-to-work processes for patients with CMD. The script was also reviewed by a physician in occupational and environmental medicine. The survey questionnaire including the script was read and commented on by three external researchers in the field, and then pilot tested with nine managers representing both men and women. Based on these pilot-tests some of the survey questions were revised, however no comments were made about the script for the vignette. For the four questions that followed the vignette, the study participants were instructed to imagine that the person in the video was their staff member/employee. The present study focuses on the first of the four questions, which relates to managers’ recommendation of sick leave.

### Study setting

In Sweden, all inhabitants of working age are covered by universal sickness insurance. The first 14 days of a sick leave spell is covered by the employer except for one qualifying day, without economic reimbursement. From day 15, sick leave benefit is granted from the Swedish Social Insurance Agency (SSIA). The employer has an obligation to plan for return to work for employees who are expected to have a sickness absence extending to 60 days.

“If it can be assumed that the insured's ability to work will be impaired due to illness for at least 60 days, the employer must, no later than the day when the insured's ability to work has been impaired for 30 days, draw up a plan for returning to work.”

(Swedish Code of Statutes 2017:1306. Chapter 30, 6 §).

If necessary, the employer/manager attends rehabilitation meetings with the employee, health care and the SSIA.

### Participants and procedure

Participants were recruited from (1) the LORE Citizen Panel at the University of Gothenburg and (2) the HELIX Competence Centre at Linköping University. The Laboratory of Opinion Research (LORE) at the University of Gothenburg conducts data collection through web-based surveys. The LORE Citizen Panel consisted of about 60,000 self-recruited individuals throughout Sweden in 2017. All the participants in LORE Citizen Panel have given an informed consent to participate in research when signing the agreement to participate (https://www.gu.se/en/som-institute/the-swedish-citizen-pan). The information to potential participants includes that the panel conducts research in different areas such as society, democracy, opinion research and health. There is also information that focused samples are used depending on the kind of research. The LORE Citizen Panel identified members who had reported that they had a managerial position by means of two questions included in the 26th Citizen Panel survey [5]. An invitation to participate in the study was sent out randomly by e-mail to 5000 of these managers 3 months later. The 5000 managers were randomized by means of the Stata statistical software and the command UniformR. The HELIX Competence Centre is a collaboration among 22 organizations (e.g. universities, private, and public organizations). Eight organizations agreed to participate and identified another 556 managers who were similarly invited. The invitation to both groups included a short introduction on the aims of the study, information about how to contact the researcher, and a question asking for consent to participate.

The survey included four questions about sick leave in relation to the video vignette. In the present study, only the first question concerning recommendation of employee sick leave was used.

### Measures

Survey questions related to the dependent variable and the independent variables are included as supplement (Additional file 1). The video case vignettes are also included as supplements (Additional files 2 and 3).

#### Dependent variable

The dependent variable was “managers’ recommendation of employee sick leave in common mental disorder vignettes”. The managers were asked: “Imagine that the woman/man in the video is your subordinate. Based on the narrative in the video, do you think that she/he needs to be granted sick leave?” The response options included “Yes, absolutely”, “Yes, probably”, “No, probably not” and “No, absolutely not”, which was dichotomized into “Yes” and “No”.

#### Independent variables

The independent variables were attitudes towards employee depression; experiences of CMD, personally or through a close friend or relative; and having previously recommended employee sick leave.

The questions on attitudes towards employee depression belong to the “Managerial stigma towards employee depression” instrument measuring managers’ affective, cognitive and behavioural attitudes to employees with depression. It is one of few questionnaires addressing managers, and was developed in Australia [10, 25]. The questionnaire was translated and culturally adapted to Swedish by our research group (of which two, MB and GH, are co-authors of this study) in close discussion with two managers and one human resources specialist. Item number 12 was changed slightly due to Swedish employment regulations from “I would try to get rid of an employee with depression” to “I would like to get rid of an employee with depression”. The questionnaire was piloted with nine Swedish managers, back translated to English by a professional translator and approved by the original author, Angela Martin. It is a 12-item scale (score range, 12–72) with higher scores representing a higher level of negative attitudes. The scale items are rated from 1 (strongly disagree) to 6 (strongly agree).

To assess experiences of CMD, personally or through a close friend or relative, managers were asked “Have you personally, or has a close relative or a friend, had depression and/or anxiety disorder?” The response options were “Yes” (1) or “No” (2).

The question on previously having recommended employee sick leave was “During the past two years, have you encouraged any staff members with depression or anxiety at your current workplace to go on sick leave?” The response options were “Yes, several staff members”, “Yes, one staff member”, and “No”. This was categorized as (1) “No”, (2) “Yes, one staff member”, and (3) “Yes several staff members”.

#### Covariates

We used managers’ person-, work- and competence-related variables as covariates. Three person-related covariates (“gender”, “age” and “level of education”) of the managers were included in the analyses. The response options for gender were “women”, “men”, and “non-binary”. Gender was dichotomized into “men” and “women,” because only a small number of respondents indicated non-binary (*n* = 3), meaning that we were unable to derive statistical inferences for this group. The options for age were “younger than 20 years”, “20–29 years”, “30–39 years”, 40–49 years”, “50–59 years”, “60–65 years” or “older than 65 years”. Based on the distribution of the responses, these options were dichotomized into " ≤ 49 years" and "50 ≥ years”. Level of education included five response options: “compulsory school”, “upper secondary school or equivalent”, “degree from college/university (minimum three years)”, and “other post-secondary education”. For the analyses, this was dichotomized into “secondary school or lower” and “post-secondary school”.

Three competence-related covariates were included in the analyses: “total years of managerial work experience”, “having had management training on CMDs”, and “having worked in an occupation treating people with CMD”. Response options for total years of managerial work experience were “0–2 years”, “3–5 years”, “6–10 years”, “ > 10 years”. based on the distribution in these categories, this variable was categorized into “0–10 years” or “ > 10 years.” Response options for having had management training on CMDs included “Yes, during the last two years”, “Yes, more than two years ago”, and “No”, which was dichotomized into “Yes” and “No”. The answer options for “During your professional life, have you worked in occupations where you cared for or treated people with depression and/or anxiety disorders?” were “Yes” or “No”.

Four work-related characteristics were also included in the analyses: “work sector”, “industries”, “size of company”, and “composition of staff by gender”. Work sector had five selection options: “governmental”, “municipal”, “country council/regional”, “private” and “non-profit organization”. These were dichotomized into “public and non-profit sector” and “private sector”. Industry was assessed with the question “in which industry does the company’s/organization’s main activity belong?”. In accordance with the Swedish Standard Industrial Classification (SNI) (https://www.scb.se/en/documentation/classifications-and-standards/swedish-standard-industrial-classification-sni/), 16 different industries were clustered into three categories according to the people-data-things hierarchy [26]. “Blue collar” refers to industries working with things, “white collar” refers to industries working with data, and “pink collar” refers to industries working with people. A fourth category "other type" was used for those industries not fitting into any of the three categories. A question on the total number of employees in the organization was used to represent the size of the company. The response options “0–9”, “10–49”, “50–250” were grouped into “0–250”, and “251–1000” and “more than 1000” into “ > 251” and “ < 250”. “Composition of staff by gender” was assessed using three response options: “most are women”, “there are about as many women as men”, and “most are men”, which were dichotomized into the response options “as many women as men”, “most are men” and “mostly women”.

Finally, the gender of the protagonist in the CMD-labelled vignette was included in the analysis.

### Statistical analyses

The Mann–Whitney U test and the chi-squared test were used to investigate associations between the dependent variable “managers’ recommendation of sick leave based on the vignettes” and the independent variables: (1) negative attitudes towards CMD, (2) experience of persons with CMD (own experience/friend/family) and (3) previous experience of recommending sick leave for staff members/employees with CMD. Associations between the dependent variable and the covariates were analysed by means of the chi-squared test.

Binary logistic regression analyses were conducted separately to generate odds ratios (ORs) with a 95% confidence interval (95% CI) for each independent variable. The multivariate analyses were conducted when the covariates were included.

Missing values were reported for every variable in descriptive analyses and reported for each regression analysis separately. All analyses were performed with IBM SPSS Statistics 26 (IBM Corp., Armonk, NY).

## Results

**Fig. 1:**
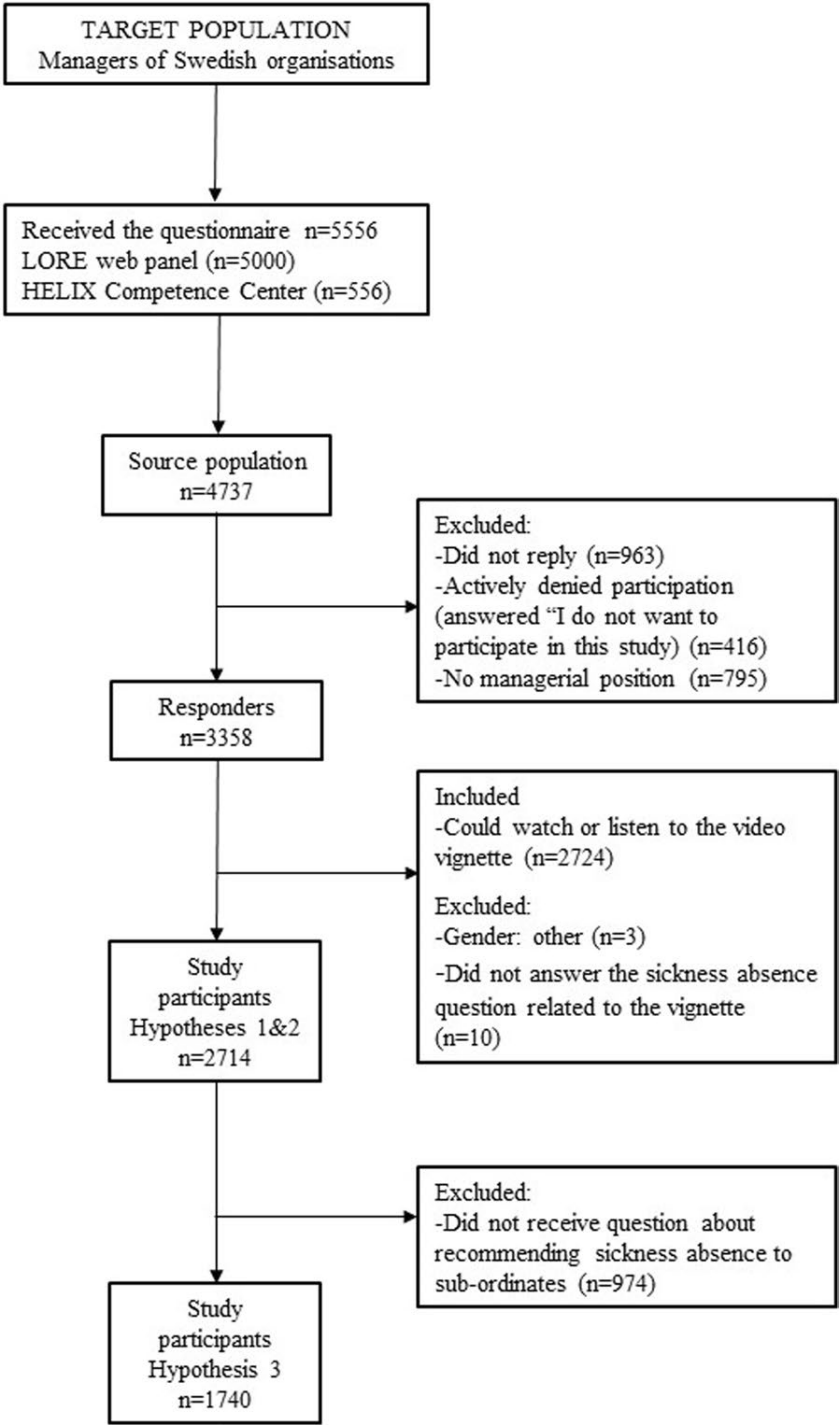
Flowchart for selection of the study population

In total, the survey was sent to 5556 prospective participants of which 3358 responded. Of those 3358, we included those who confirmed that they had been watched the video vignette (*n* = 2724). Of those, ten did not answer the question about recommending sick leave, resulting in 2714 study participants. For hypothesis 3, we used a subsample (n = 1740) because the question on earlier experience of recommending sick leave was only shown to participants who confirmed having had subordinates with CMD in the last 2 years. A flowchart showing the selection from the target population to the study sample and subsample is presented in Fig. 1 (Additional file 4).

### Characteristics of the study participants

The characteristics of the participants are presented in Table 1 (Additional file 5). The table also compares the proportions regarding these characteristics between the study population (*n* = 2714) and the subsample (*n* = 1740).

**Table 1:** The characteristics of the participants and comparisons in proportions regarding these characteristics between the study population (*N* = 2714) and the subsample (*n* = 1740)

Characteristics of the participants	Study sample (n = 2714)		Subsample (*n* = 1740)^a^		95% confidence interval^b^
	n	%	n	%	
*Personal-related characteristics*					
*Gender*					
Women	929	34.2	676	38.9	(**− 0.075 to − 0.017**)
Men	1774	65.4	1056	60.7	
Missing	11		8		
*Age*					
≤50 years	1400	51.6	900	51.7	(− 0.031 to 0.029)
≥51 years	1314	48.4	840	48.3	
Missing	0		0		
*Level of education*					
Secondary school or less	393	14.5	235	13.5	(− 0.011 to 0.031)
Post-secondary	2321	85.5	1505	86.5	
Missing	0		0		
*Competence-related characteristics*					
*Years of managerial work experience*					
0–10 years	1348	49.7	865	49.7	(− 0.031 to 0.03)
>10 years	1366	50.3	875	50.3	
Missing	0		0		
*Having had management training on CMDs*					
Yes	727	26.8	502	28.9	(− 0.048 to 0.006)
No	1987	73.2	1238	71.1	
Missing	0		0		
*Having been in occupations treating or caring for people with CMDs*					
Yes	441	16.3	322	18.6	(**− 0.045 to 0.0**)
No	2261	83.7	1409	81.4	
Missing	12		9		
*Work-related characteristics*					
Sector					
Private	1552	57.2	888	48.9	(**0.032 − 0.091**)
Public or non-profit	1160	42.7	851	51	
Missing	2		1		
*Industry*					
Blue collar	825	30.4	511	29.4	(− 0.017 to 0.038)
White collar	612	22.5	330	19	(**0.012 − 0.06**)
Pink collar	842	31	622	35.7	(**− 0.076 to − 0.019**)
Other	435	16	277	15.9	(− 0.021 to 0.023)
Missing	0		0		
Number of employees in organization					
0–250	1368	50.4	835	48	(− 0.006 to 0.054)
>250	1346	49.6	905	52.3	
Missing	0		0		
C*omposition of staff by gender*					
Mostly male	1758	64.8	1058	60.8	(**0.011 − 0.069**)
Mostly female	956	35.2	682	39.2	
Missing	0		0		
*Attitude to CMDs*					
No negative attitude (score 12–35)	2121	79.6	1414	82.8	(**− 0.055 to − 0.007**)
Negative attitude (score 36+)	543	20.4	294	17.2	
Missing	50		32		
*Personal experience of CMD*					
Yes	2076	76.8	1371	79.2	(− 0.048 to 0.002)
No	628	23.2	361	20.8	
Missing	10		8		
Having recommended sick leave to employee					
No			1011	58.2	Not applicable
Yes, to one employee			565	32.5	
Yes, to several employees			161	9.3	
Missing			3		

Values in bold are significant

^a^Subsample consisted of those study participants who answered yes to the question of having experience of one or several subordinates with CMD, because only those participants got the question about having recommended sick leave to a subordinate

^b^95% Confidence intervals were calculated for the difference in proportions between the study sample and the subsample for each characteristic

### Associations between the dependent and the independent variables

Of those study participants with negative attitudes towards employee depression, 62% recommended sick leave and 38% did not recommend sick leave regarding the CMD vignettes. The results showed that there was no significant association (*p* = 0.071) between negative attitudes towards employee depression and managers’ recommendation of employee sick leave for the CMD vignettes. Thus, this variable was not included in further analyses.

The results showed that managers’ experience with CMDs, personally or through a close friend or relative, was significantly associated (*p* = 0.047) with managers’ recommendation of employee sick leave for the CMD vignettes.

The results showed that having previously recommended employee sick leave to one employee was significantly associated (*p* < 0.001) with managers’ recommendation of employee sick leave for the CMD vignettes. Furthermore, previously having recommended employee sick leave to several employees was significantly associated (*p* < 0.001) with managers’ recommendation of employee sick leave for the CMD vignettes.

### Associations between the dependent variables and the covariates

Two person-related covariates showed a significant association with the dependent variable (managers’ recommendation of sick leave with regard to the video vignettes): gender (male managers significantly more often than female managers; *p* = 0.005) and level of education (managers with higher education significantly more often than managers with lower education; *p* = 0.001). Age was not significantly associated with managers’ recommendation of sick leave (*p* = 0.051).

Two competence-related covariates showed a significant association with the dependent variable: total years of managerial work experience (*p* < 0.001) and having had management training on CMD (*p* = 0.031). Having worked in an occupation treating people with CMD was not significantly associated with managers’ recommendation of sick leave (*p* = 0.067).

None of the work-related covariates showed a significant association with the dependent variable as shown by the p values ranging between 0.201 and 0.583.

Finally, the gender of the protagonist in the vignette was not significantly associated with managers’ recommendation of sick leave (*p* = 0.991).

### Determinants of managers’ recommendation of sick leave

#### Managers with experience of CMD

The results of the bivariate and multivariate binary logistic regression are presented in Table 2 (Additional file 6). A bivariate analysis yielded a significant association between managers with experience of CMD and managers’ recommendation of employee sick leave based on the CMD video vignettes (Model 1). In the second and final model (Model 2), which adjusted for the person-related covariates, the odds became insignificant (*p* = 0.056).

**Table 2:** Crude and adjusted odds ratios (OR) with 95% confidence interval (CI) for “Managers recommending sick leave based on the video vignettes” with respect to the managers’ experience of CMD

Independent variable	Managers recommending sick leave based on the video vignettes		
	*n*	Model 1: crude OR (95% CI)	Model 2: OR (95% CI)
Managers without experience of CMD	628	**1**	**1**
Managers with experience of CMD (yes)	2076	**0.8 (0.70–0.98**)	1.2 (1.00–1.44)

Values in bold are significant. *n*, number of cases in the regression models; missing cases, 10

Model 1, bivariate analyses; Model 2, final model adjusted for personal-related characteristics (gender, level of education)

As the results changed after adjusting for gender and educational level (Table 2), stratified analyses were performed for men, women, higher educational level and lower educational level. None of the results from these analyses showed significant *p* values.

#### Managers previous recommendation on sick leave for one or more employees

The results of the bivariate and multivariate binary logistic regression are presented in Table 3 (Additional file 7). A bivariate analysis yielded a significant association between managers with experience of recommending sick leave to one employee and to several employees, and managers’ recommendation of employee sick leave for CMD video vignettes (Model 1). Separate multivariate analyses adjusting for the covariates were performed (Models 2–4). When entering the person-related covariates, the odds remained significant, only slightly attenuated (Model 2). However, when entering the competence-related covariates, the ORs remained significant and increased (Model 3). In the final model, the ORs remained significant (Model 4).

**Table 3:** Crude and adjusted odds ratios (OR) with 95% confidence interval (CI) for “Managers recommending sick leave based on the video vignettes” with respect to managers’ previous experience of recommending sick leave in real life

Independent variable	Managers recommending sick leave based on the video vignettes				
	*n*	Model 1: crude OR (95% CI)	Model 2: OR (95% CI)	Model 3: OR (95% CI)	Model 4: OR (95% CI)
Managers without previous experience of recommending sick leave to employee	1006	**1**	**1**	**1**	**1**
Managers with previous experience of recommending sick leave to one employee (yes)	564	**3.7 ** **(2.90–4.69)**	**3.6 ** **(2.84–4.62)**	**4.0 ** **(3.11–5.21)**	**3.7 (** **2.90–4.73)**
Managers with previous experience of recommending sick leave to several employees (yes)	159	**7.0 ** **(4.21–11.58)**	**6.8 ** **(4.10–11.29)**	**7.9 ** **(4.65–13.60)**	**7.1 ** **(4.27–11.82)**

Values in bold are significant. *n*, number of cases included in the regression models; missing cases, 11. Model 1, bivariate analyses; Model 2, model adjusted for personal-related characteristics (gender, level of education); Model 3, model adjusted for competence-related characteristics (total years of managerial work experience, having had management training on CMDs); Model 4. fully adjusted model

## Discussion

This cross-sectional study included 2714 Swedish managers drawn from a variety of work sectors and industries. The results showed that managers’ previous experience of recommending sick leave to employee/employees were strong determinants for recommending sick leave with regard to the CMD-related cases shown in the video vignettes. The likelihood of a manager recommending sick leave after watching a CMD-labelled video vignette was higher if the manager had previous experience of this situation in real life. Thus, these results confirmed our third hypothesis, i.e. that managers with previous experience of recommending sick leave for people with CMD would recommend sick leave to a significantly higher extent also based on the vignettes. In contrast, however, the results did not confirm our first and second hypotheses regarding managers’ attitudes towards employees with CMD and managers’ experiences of CMD, personally or through a close friend or relative.

Studies regarding determinants of managerial behaviour in relation to recommending sickness absence are scarce. Our study seems to be the first to explore managers’ previous experience of recommending employee sick leave and the recommendation of employee sick leave based on video vignettes, while also considering managers’ personal characteristics, manager competence and workplace factors. Thus, our study is likely the first to find that managers’ previous experience of recommending sick leave to employees with CMD may be a predictor of recommending employee sick leave at the workplace.

Managers’ behaviour regarding recommending sick leave based on the video vignettes may not necessarily be interpreted as right or wrong. Managers may have an individual or an organizational perspective on the need for employee sick leave, or a combination of those. Factors with an individual perspective that argue for recommending sick leave may include managers’ concerns about employee mental health deterioration and the need for rest and recuperation. From an organizational perspective, factors arguing for recommending sick leave may include managers’ concerns about employees’ decreased work capacity in relation to general errors, specific errors, third-party safety, or productive issues in the workplace. All these factors have been found to be associated with decreased work capacity among employees with CMD [27]. Research exploring physicians’ decision-making of whether to issue sickness absence or not showed that the safety concerns for the patient (employee), the workplace or the third party were of great importance in their decision-making process [28]. A further factor could be that the managers lack sufficient knowledge of CMD and preventive actions, and therefore recommend employee sick leave rather than exploring alternatives. In this case, managers may continue to recommend sick leave for future employees with CMD for lack of alternatives when person-centred support and preventive actions at the workplace would be more beneficial.

There is an increasing body of research showing that managerial training on CMD improves managers’ knowledge, attitudes, self-confidence and self-reported preventive actions [29–31]. In an interesting study from Australia [32], a significant reduction in work injury-related sickness absence (but not in standard sickness absence) was found after managerial training in mental health at workplaces for firefighters and rescue workers. However, studies have found that only 14–25% of managers reported having had managerial training on CMD [33]. The employers are responsible for managerial training and for support to managers on an organizational level*.* How managers’ organizations and contexts support or hamper them in the prevention of CMD-related sickness absence needs attention and further research.

Contrary to the first of hypothesis concerning attitudes towards CMD, the results showed that negative attitudes towards employees with CMD were not associated with managers’ recommendation of employee sick leave based on the video vignettes. This finding, although encouraging, warrants some explanation. The study participants’ level of education was relatively high (Table 1). Managers who have higher education are less likely to hold negative attitudes towards CMD [10]. It is also plausible that the managers who agreed to answer the survey had an interest in, and commitment to, mental health issues and therefore they held fewer negative attitudes towards CMD. In our cohort, 64% had experience of employees with CMD, and others have found that negative attitudes decreased in the relation to the increased number of employees with CMD that the managers had dealt with [13]. Moreover, in our cohort, more male managers declined participation [5], and male managers [10, 13] and men in general [34, 35] have shown more negative attitudes towards CMD. If so, managers holding negative attitudes towards CMD may be underrepresented in the study.

The second hypothesis was that managers with experience of CMD would recommend sick leave to a significantly higher extent than managers lacking this experience. Although significant in the bivariate analysis, the results of the final adjusted regression model became non-significant, thus showing that any personal experience of CMD was not associated with managers’ recommendation of employee sick leave based on the video vignettes. Individuals with CMD have shared their experiences about the difficulties in making others understand how their work capacity can be affected [23]. Likewise, managers in qualitative studies have reported their difficulties in understanding how CMD can affect work capacity and work performance among these employees [33, 36]. Managerial first-hand experience likely renders a different understanding of how CMD can affect work capacity as opposed to when the experience is related to a close relative or friends with CMD. It may be that this question was too broad because it included both personal CMD and CMD in close relatives and friends. In future research, dividing this question into one question on first-hand experience of CMD and another question concerning close relatives and friends could shed more light on this matter.

Finally, the strong association found between past and future behaviour in our study is a novel finding and suggests that this result needs further attention. How is it that managers begin to recommend their employees to go on sick leave in the first place? Do managers have alternative measures to prevent sickness absence, or is knowledge of such measures lacking?

## Strengths and limitations

The large study sample size and the inclusion of managers drawn from a variety of work sectors and industries were important strengths of the study. The samples used reflect the distribution of men and women as managers in Sweden [37]. Another strength of the study was the use of instrument to measure managers’ attitudes towards employees with CMD which has been validated in the English version [10, 25]. The Swedish version of the instrument, first used in the project of which this study is part, was tested for face validity in the translation process. In another study in the project by Mangerini et al. [13] calculated Cronbach Alpha for the Swedish version and found a coefficient of 0.80 which suggests good internal consistency. Further psychometric testing of the instrument is warranted. Furthermore, the data made it possible to control for several competence and contextual variables. The workplace context can provide constraints or opportunities that affect the occurrence of attitudes and behaviours in organizations [38]. Gender organizational structure and culture are likely to affect leadership behaviour [39]. In the analyses we controlled for gender composition of the workplace, work sector, industries, and size of company. Future studies can explore further possibilities to take organizational context into consideration when investigating managers’ attitudes and behaviour regarding employees with CMD and sick leave.

The present study’s method involved a survey with a video vignette. Case vignettes are a common method in experimental research used to explore participants’ responses to presumptive situations [17]. Video vignettes have been proposed to be more beneficial than written vignettes, for example, regarding reliability [18].

We used a female and a male protagonist to be able to control for protagonist gender in the analyses, which further strengthens our method. The setting in the video was that the person had asked for an appointment with the manager and the conversation took place in the manager’s office. Depending on the managers’ work sector (“governmental”, “municipal”, “country council/regional”, “private”, “non-profit organization”) and industry (“blue collar”, “white collar”, “pink collar”, “other type”), the video vignette might be more or less relatable and thus serve as a stronger or weaker trigger. Video vignettes adapted to industry could present a more realistic representation of managers’ real-life work situation, which could help to increase the validity and generalizability of the results. Future research on the topic should consider this.

Nevertheless, there are some limitations to the study. None of the samples were randomly selected. The Citizen Panel consists of mostly self-recruited participants and self-identified managers. A drawback of self-recruited web panels is that the results are not necessarily representative of the population at large. We included a first question “are you a manager”, but still, self-identification as managers might introduce bias of participants who are not managers. In contrast, the HELIX sample comprises employer-identified managers, a more valid sample, but with a low participation rate similar to other studies [8]. We chose to use these two samples, with their drawbacks, as a cost-effective way to reach managers. There are no available registers or databases of managers in Sweden.

Among the sample from the Citizen Panel (70% of the non-responders), more men refrained from participation and more managers aged 50–65 years participated compared with the dropouts. This means that the sample is biased, as mentioned earlier, towards more well-educated participants and with a higher proportion of women. Both factors will lead to a probable under-representation of persons with negative attitudes. However, this study mainly assessed associations, and there were enough participants with both negative and positive attitudes to show an association with how sick leave is recommended had there been one.

Unfortunately, it is not possible to assess the distribution of non-participation in the HELIX sample.

Regarding internal attrition among the 3358 managers, 7% did not answer any item on the Managerial stigma towards employee depression (MSED) scale. It might be that the non-responders and the attrition in the MSED have contributed to a smaller number of negative attitudes among the participants; however, that would affect proportions but not associations. The vignettes were placed after the MSED, and the inclusion of a filmed vignette caused a large internal attrition; 19% had not seen or answered the question on whether they had seen the vignette or not. Nevertheless, of those who could watch the video vignette (*n* = 2724), only ten participants did not answer the following question about recommending sick leave. To watch a filmed vignette necessitates suitable equipment and surroundings. Unfortunately, we did not inform the participants in the introduction to the survey that it contained a video vignette. Future surveys applying the same method should consider including this information at the beginning so that the respondents are prepared, and they can solve any technical issues or chose an environment better suited for listening to and watching a video. Future studies could also add sub-text to video vignettes to support listening and comprehension in case of a disturbing environment.

With regard to integrity and the project aim (employees with CMD), the question about personal experience of CMD (Hypothesis 2) was broad including personal CMD and that of close relatives and friends. That might have introduced a mismatch of experiences, which could have affected the results.

## Conclusion

This study found that the likelihood of a manager recommending sick leave after watching a video vignette of an imaginary employee with CMD was higher if the manager had done so earlier in his/her work as manager. This association remained significant after controlling for covariates. The study found no association between attitudes towards depression or personal experience of CMD and recommending sick leave after watching a video vignette. We consider this study important as a first study looking into managers’ attitudes and personal experience and recommendations of sick leave. Few studies focus on the earliest part of the sickness absence process. This study highlights the importance of including managerial behaviours and attitudes to better understand sick leave among employees with CMD. Future studies should go further and vary the presentations of video vignettes, so they adapt more to the specificities of the managers’ work sectors.

## Supplementary Information

**Additional file 1** Questions from the eu35_manager online survey used in the study.

**Additional file 2** Video vignette female.

**Additional file 3** Video vignette male.

**Additional file 4** Figure S1. Flowchart for selection of the study population.

**Additional file 5** Table S1. The characteristics of the participants and comparisons in proportions regarding these characteristics between the study population (N=2714) and the subsample (n=1740).

**Additional file 6** Table S2. Crude and adjusted odds ratios (OR) with 95% confidence interval (CI) for “Managers recommending sick leave based on the video vignettes” with respect to the managers’ experience of CMD.

**Additional file 7** Table S3, Crude and adjusted odds ratios (OR) with 95% confidence interval (CI) for “Managers recommending sick leave based on the video vignettes” with respect to managers’ previous experience of recommending sick leave in real life.

## Data Availability

The data used for this study are archived at the Laboratory of Opinion Research (LORE) at the University of Gothenburg and can be obtained by contacting LORE at info@lore.gu.se. The setting in the video vignette was that the person had asked for an appointment with the manager and the conversation took place in the manager’s office. The protagonists then shared their problems in handling their work situation with the manager. Professional actors played the video vignettes, one female and one male. The video vignettes are included as an appendix.

## Abbreviations

CMD: Common mental disorders
MSED: Managerial stigma towards employee depression
SSIA: Swedish Social Insurance Agency
