# Supplementary material for: Managers’ sick leave recommendations for employees with common mental disorders: a cross-sectional video vignette study
Source: BMC Psychol. 2023 Feb 24;11:52. doi: 10.1186/s40359-023-01086-6 (PMC9951527; doi:10.1186/s40359-023-01086-6)
Supplement: Supplementary file 1 — Additional file 1 Questions from the eu35_manager online survey used in the study. [file 40359_2023_1086_MOESM1_ESM.docx]

# Questions from the eu35_manager online survey

q36 On the next page you will see a two-minute video of a person describing a work situation for the person’s boss/manager. Then you will have a chance to answer some questions about how you assess the person’s work situation.

Please check to ensure that you have sound enabled on the device that you are using to respond to the questionnaire.

Q39 Watch the following video link and then answer the subsequent questions: (vignette with a man/woman)

q43 Imagine that the man/woman in the video is your staff member. Based on the narrative in the video, do you think that he/she needs to be granted sick leave?

o Yes, absolutely (1)

o Yes, probably (2)

o No, probably not (3)

o No, absolutely not (4)

q51 We will ask a number of questions here about your experience with depression and anxiety disorders.

q52 During the past two years, have you had staff members at your current workplace who have had depression and/or anxiety disorders?

The question also applies to those who have worked less than two years at the current workplace.

o Yes, several staff members (1)

o Yes, one staff member (2)

o No, no staff member (3)

o Don't know (4)

q54 During the past two years, have you encouraged any staff members with depression or anxiety at your current workplace to go on sick leave?

o Yes, several staff members (1)

o Yes, one staff member (2)

o No (3)

q65 Have you personally, or has a close relative or a friend, had depression and/or anxiety disorders?

o Yes (1)

o No (2)

The questions on attitudes towards employee depression belong to the “Managerial stigma towards employee depression” instrument measuring managers’ affective, cognitive and behavioural attitudes to employees with depression. References:

Martin A. Individual and contextual correlates of managers' attitudes toward depressed employees. Hum Resour Manage. 2010;49(4):647–668.

Martin AJ, Giallo R. Confirmatory factor analysis of a questionnaire measure of managerial stigma towards employee depression. Stress Health. 2016;32(5):621–628

Mangerini I, Bertilsson M, de Rijk A, Hensing G. Gender differences in managers’ attitudes towards employees with depression: a cross-sectional study in Sweden. BMC Public Health. 2020;20(1):1–15.
