# Supplementary material for: Managers’ sick leave recommendations for employees with common mental disorders: a cross-sectional video vignette study
Source: BMC Psychol. 2023 Feb 24;11:52. doi: 10.1186/s40359-023-01086-6 (PMC9951527; doi:10.1186/s40359-023-01086-6)
Supplement: Supplementary file 4 — Additional file 4 Figure S1. Flowchart for selection of the study population. [file 40359_2023_1086_MOESM4_ESM.docx]

Figure 1. Flowchart for selection of the study population
