# Supplementary material for: Managers’ sick leave recommendations for employees with common mental disorders: a cross-sectional video vignette study
Source: BMC Psychol. 2023 Feb 24;11:52. doi: 10.1186/s40359-023-01086-6 (PMC9951527; doi:10.1186/s40359-023-01086-6)
Supplement: Supplementary file 5 — Additional file 5 Table S1. The characteristics of the participants and comparisons in proportions regarding these characteristics between the study population (N=2714) and the subsample (n=1740). [file 40359_2023_1086_MOESM5_ESM.docx]

**Table 1** The characteristics of the participants and comparisons in proportions regarding these characteristics between the study population (*N*=2714) and the subsample (*n*=1740)

| Characteristics of the participants | Study sample (n=2714) | | Subsample (*n*=1740)^a^ | | 95% confidence interval^b^ |
| --- | --- | --- | --- | --- | --- |
|  | n | % | n | % |  |
| Personal-related characteristics | | | | | |
| Gender |  |  |  |  |  |
| Women | 929 | 34.2 | 676 | 38.9 | (**−0.075 to −0.017**) |
| Men | 1774 | 65.4 | 1056 | 60.7 |  |
| Missing | 11 |  | 8 |  |  |
| Age |  |  |  |  |  |
| ≤50 years | 1400 | 51.6 | 900 | 51.7 | (−0.031 to 0.029) |
| ≥51 years | 1314 | 48.4 | 840 | 48.3 |  |
| Missing | 0 |  | 0 |  |  |
| Level of education |  |  |  |  |  |
| Secondary school or less | 393 | 14.5 | 235 | 13.5 | (−0.011 to 0.031) |
| Post-secondary | 2321 | 85.5 | 1505 | 86.5 |  |
| Missing | 0 |  | 0 |  |  |
| Competence-related characteristics | | | | | |
| Years of managerial work experience |  |  |  |  |  |
| 0–10 years | 1348 | 49.7 | 865 | 49.7 | (−0.031 to 0.03) |
| >10 years | 1366 | 50.3 | 875 | 50.3 |  |
| Missing | 0 |  | 0 |  |  |
| Having had management training on CMDs |  |  |  |  |  |
| Yes | 727 | 26.8 | 502 | 28.9 | (−0.048 to 0.006) |
| No | 1987 | 73.2 | 1238 | 71.1 |  |
| Missing | 0 |  | 0 |  |  |
| Having been in occupations treating or caring for people with CMDs |  |  |  |  |  |
| Yes | 441 | 16.3 | 322 | 18.6 | (**−0.045 to 0.0**) |
| No | 2261 | 83.7 | 1409 | 81.4 |  |
| Missing | 12 |  | 9 |  |  |
| Work-related characteristics | | | | | |
| Sector |  |  |  |  |  |
| Private | 1552 | 57.2 | 888 | 48.9 | (**0.032−0.091**) |
| Public or non-profit | 1160 | 42.7 | 851 | 51 |  |
| Missing | 2 |  | 1 |  |  |
| Industry |  |  |  |  |  |
| Blue collar | 825 | 30.4 | 511 | 29.4 | (−0.017 to 0.038) |
| White collar | 612 | 22.5 | 330 | 19 | (**0.012−0.06**) |
| Pink collar | 842 | 31 | 622 | 35.7 | (**−0.076 to −0.019**) |
| Other | 435 | 16 | 277 | 15.9 | (−0.021 to 0.023) |
| Missing | 0 |  | 0 |  |  |
| Number of employees in organization |  |  |  |  |  |
| 0–250 | 1368 | 50.4 | 835 | 48 | (−0.006 to 0.054) |
| >250 | 1346 | 49.6 | 905 | 52.3 |  |
| Missing | 0 |  | 0 |  |  |
| Composition of staff by gender |  |  |  |  |  |
| Mostly male | 1758 | 64.8 | 1058 | 60.8 | (**0.011−0.069**) |
| Mostly female | 956 | 35.2 | 682 | 39.2 |  |
| Missing | 0 |  | 0 |  |  |
| Attitude to CMDs |  |  |  |  |  |
| No negative attitude (score 12–35) | 2121 | 79.6 | 1414 | 82.8 | (**−0.055 to −0.007**) |
| Negative attitude (score 36+) | 543 | 20.4 | 294 | 17.2 |  |
| Missing | 50 |  | 32 |  |  |
| Personal experience of CMD |  |  |  |  |  |
| Yes | 2076 | 76.8 | 1371 | 79.2 | (−0.048 to 0.002) |
| No | 628 | 23.2 | 361 | 20.8 |  |
| Missing | 10 |  | 8 |  |  |
| Having recommended sick leave to employee |  |  |  |  |  |
| No |  |  | 1011 | 58.2 | Not applicable |
| Yes, to one employee |  |  | 565 | 32.5 |  |
| Yes, to several employees |  |  | 161 | 9.3 |  |
| Missing |  |  | 3 |  |  |

Values in bold are significant.

^a^Subsample consisted of those study participants who answered yes to the question of having experience of one or several subordinates with CMD, because only those participants got the question about having recommended sick leave to a subordinate.

^b^95% Confidence intervals were calculated for the difference in proportions between the study sample and the subsample for each characteristic.
