# Supplementary material for: Managers’ sick leave recommendations for employees with common mental disorders: a cross-sectional video vignette study
Source: BMC Psychol. 2023 Feb 24;11:52. doi: 10.1186/s40359-023-01086-6 (PMC9951527; doi:10.1186/s40359-023-01086-6)
Supplement: Supplementary file 6 — Additional file 6 Table S2. Crude and adjusted odds ratios (OR) with 95% confidence interval (CI) for “Managers recommending sick leave based on the video vignettes” with respect to the managers’ experience of CMD. [file 40359_2023_1086_MOESM6_ESM.docx]

**Table 2** Crude and adjusted odds ratios (OR) with 95% confidence interval (CI) for “Managers recommending sick leave based on the video vignettes” with respect to the managers’ experience of CMD

| Independent variable | Managers recommending sick leave based on the video vignettes | | |
| --- | --- | --- | --- |
|  | *n* | Model 1: crude OR (95% CI) | Model 2: OR (95% CI) |
| Managers without experience of CMD | 628 | **1** | **1** |
| Managers with experience of CMD (yes) | 2076 | **0.8 (0.70–0.98**) | 1.2 (1.00–1.44) |

Values in bold are significant. *n*, number of cases in the regression models; missing cases, 10.
Model 1, bivariate analyses; Model 2, final model adjusted for personal-related characteristics (gender, level of education).
