# Supplementary material for: Managers’ sick leave recommendations for employees with common mental disorders: a cross-sectional video vignette study
Source: BMC Psychol. 2023 Feb 24;11:52. doi: 10.1186/s40359-023-01086-6 (PMC9951527; doi:10.1186/s40359-023-01086-6)
Supplement: Supplementary file 7 — Additional file 7 Table S3, Crude and adjusted odds ratios (OR) with 95% confidence interval (CI) for “Managers recommending sick leave based on the video vignettes” with respect to managers’ previous experience of recommending sick leave in real life. [file 40359_2023_1086_MOESM7_ESM.docx]

**Table 3** Crude and adjusted odds ratios (OR) with 95% confidence interval (CI) for “Managers recommending sick leave based on the video vignettes” with respect to managers’ previous experience of recommending sick leave in real life

| Independent variable | Managers recommending sick leave based on the video vignettes | | | | |
| --- | --- | --- | --- | --- | --- |
|  | *n* | Model 1: crude OR (95% CI) | Model 2: OR (95% CI) | Model 3: OR (95% CI) | Model 4: OR (95% CI) |
| Managers without previous experience of recommending sick leave to employee | 1006 | **1** | **1** | **1** | **1** |
| Managers with previous experience of recommending sick leave to one employee (yes) | 564 | **3.7  (2.90–4.69)** | **3.6  (2.84–4.62)** | **4.0  (3.11–5.21)** | **3.7 ( 2.90–4.73)** |
| Managers with previous experience of recommending sick leave to several employees (yes) | 159 | **7.0  (4.21–11.58)** | **6.8  (4.10–11.29)** | **7.9  (4.65–13.60)** | **7.1  (4.27–11.82)** |

Values in bold are significant. *n*, number of cases included in the regression models; missing cases, 11. Model 1, bivariate analyses; Model 2, model adjusted for personal-related characteristics (gender, level of education); Model 3, model adjusted for competence-related characteristics (total years of managerial work experience, having had management training on CMDs); Model 4. fully adjusted model.
